# An Evolutionary Hypothesis of Binary Opposition in Functional Incompatibility about Habenular Asymmetry in Vertebrates

**DOI:** 10.3389/fnins.2016.00595

**Published:** 2017-01-04

**Authors:** Hiroyuki Ichijo, Tomoya Nakamura, Masahumi Kawaguchi, Yuichi Takeuchi

**Affiliations:** Department of Anatomy and Neuroscience, Graduate School of Medicine and Pharmaceutical Sciences, University of ToyamaToyama, Japan

**Keywords:** scale-eating, zebrafish, mouse, habenula, asymmetry, lateralization, natural selection

## Abstract

Many vertebrates have asymmetrical circuits in the nervous system. There are two types of circuit asymmetry. Asymmetrical circuits in sensory and/or motor systems are usually related to lateralized behaviors. It has been hypothesized that spatial asymmetry in the environment and/or social interactions has led to the evolution of asymmetrical circuits by natural selection. There are also asymmetrical circuits that are not related to lateralized behaviors. These circuits lie outside of the sensory and motor systems. A typical example is found in the habenula (Hb), which has long been known to be asymmetrical in many vertebrates, but has no remarkable relationship to lateralized behaviors. Instead, the Hb is a hub wherein information conveyed to the unilateral Hb is relayed to diverging bilateral nuclei, which is unlikely to lead to lateralized behavior. Until now, there has been no hypothesis regarding the evolution of Hb asymmetry. Here, we propose a new hypothesis that binary opposition in functional incompatibility applies selection pressure on the habenular circuit and leads to asymmetry. Segregation of the incompatible functions on either side of the habenula is likely to enhance information processing ability via creating shorter circuits and reducing the cost of circuit duplication, resulting in benefits for survival. In zebrafish and mice, different evolutionary strategies are thought to be involved in Hb asymmetry. In zebrafish, which use a strategy of structurally fixed asymmetry, the asymmetrical dorsal Hb leads to constant behavioral choices in binary opposition. In contrast, in mice, which use a strategy of functionally flexible lateralization, the symmetrical lateral Hb is functionally lateralized. This makes it possible to process complicated information and to come to variable behavioral choices, depending on the specific situation. These strategies are thought to be selected for and preserved by evolution under selection pressures of rigidity and flexibility of sociability in zebrafish and mice, respectively, as they are beneficial for survival. This hypothesis is highly valuable because it explains how the Hb evolved differently in terms of asymmetry and lateralization among different species. In addition, one can propose possible experiments for the verification of this hypothesis in future research.

## Introduction

Brain asymmetry and functional lateralization are recognized in many vertebrates. It is well-known that the left side of the cerebral hemisphere is dominant in language in most humans, especially in right-handed persons (Wada and Rasmussen, 1960; McGlone, 1984). Higher brain functions are differentiated based on brain hemisphere. Brain lateralization has been experimentally analyzed. The results of such experiments indicate that social cognition and interactions between predator and prey are involved in lateralized animal behaviors. The most well-known example of lateralized behavior in a neuronal circuit is found in birds, especially in the chick visual system (Rogers and Anson, 1979; Rogers, 1982, 2000; Evans et al., 1993; Rogers and Andrew, 2002). At the behavioral level, the chick utilizes the left and right eyes differently, depending on the situation. Chicks tilt their heads, using mainly their left eye to see the sky. They thus use the left eye for detecting flying predators. The left eye and the downstream visual circuits, which cross to the right side of the optic tectum, are more sensitive to moving objects. On the other hand, chicks see the ground mainly using their right eye, which is used for searching for feed on the ground. The right eye and the left optic tectum are more sensitive to fine features. Lateralization of the visual system enables chicks to perform different tasks independently and simultaneously. Lateralized circuits make parallel processing of different visual information possible and increase the probability of survival for the chick. Therefore, lateralized behavior is naturally selected for and implemented in the structures of the asymmetrical circuits.

**Figure 1:**
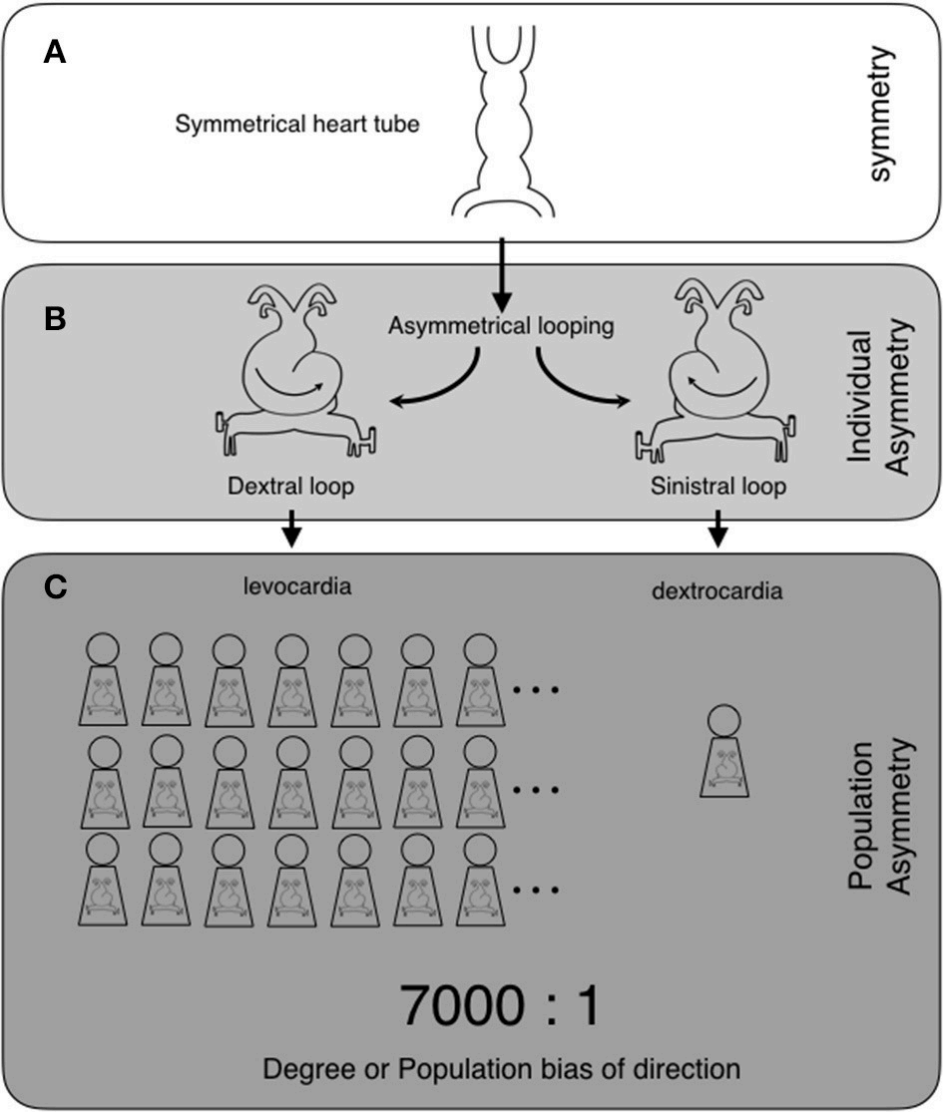
**Two levels of asymmetry: individual asymmetry and population asymmetry, and their examples in heart formation**. The heart tube is originally symmetrical **(A)**. The heart tube undergoes asymmetrical morphogenesis. This consists of dextral or sinistral looping, which makes the heart asymmetrical. Dextral looping causes levocardia, where the heart lies on the left side of the thorax. On the other hand, sinistral looping causes dextrocardia, where the heart lies on the right (individual asymmetry) **(B)**. In human populations, almost all individuals have levocardia (population asymmetry), with the direction of the asymmetry toward the left side. Coinciding with situs inversus, dextrocardia occurs in about 1/7000 individuals. Thus, the heart exhibits asymmetry with a population bias of 7000:1 (levocardia vs. dextrocardia) (“degree of direction” or “population bias of direction”) **(C)**.

Before discussing asymmetry at different levels, we will define the following terms: individual asymmetry, population asymmetry, and direction of asymmetry. We will explain these terms using examples of a well-known asymmetrical structure, the heart, which is a part of the cardiovascular system. In the beginning of heart formation, a symmetrical heart tube undergoes dextral looping, resulting in an S-shaped heart tube and leading to the formation of an asymmetrical heart (Figures 1A,B). The dextral looping causes levocardia, which causes the heart to lie on the left side of the thorax. Therefore, the heart is asymmetrical in all individuals. “Individual asymmetry” means that the left and the right sides are asymmetrically different in individuals. In almost all individuals in the human population, the heart tube undergoes dextral looping. However, in rare cases, it undergoes looping in the opposite direction. Sinistral looping causes dextrocardia, which is when the heart lies on the right. “Population asymmetry” means that individuals with hearts on one or the other side are more predominant in the population (Figure 1C). “Direction of asymmetry” represents the predominant side. Coinciding with situs inversus, in which the visceral organs are mirrored from their normal positions, dextrocardia occurs in about 1/7000 individuals (Sadler, 2004). The ratio of the left- or right-sidedness of the organs in the population is represented by the “degree of the direction” or the “population bias of the direction.” Thus, the heart exhibits an asymmetry of levocardia or dextrocardia with a population bias of 7000:1, which indicates that heart asymmetry is highly biased toward the left.

In the nervous system, symmetry breaking is usually observed in two ways: structures and behavior. Thus, the words asymmetry and lateralization are often used differentially. Considering the links between the structures/functions of circuits and behavior, it is understandable that asymmetrical structures/functions of neuronal circuits direct lateralized behaviors, which interact with the environment in an asymmetric manner. Even when circuits are structurally symmetrical, they may be used asymmetrically. This results in functional lateralization, which can lead to lateralized behavior. Thus, we need to discriminate between structural asymmetry and functional lateralization when we analyze behavioral lateralization. In general, the word “asymmetry” is usually used to describe structures and “lateralization” is used to describe functions and/or behaviors. Functional and behavioral lateralizations are similar, but are not exactly equivalent. For example, in mice, the Hb is lateralized, but there is no remarkable behavioral lateralization (Ichijo et al., 2015). It is also logically possible that asymmetrically structured circuit is functionally lateralized in a manner that leads to no lateralized behavior. Because functional lateralization does not necessarily generate lateralized behavior, we will use the terms lateralization of function and lateralization of behavior as terms with distinct meanings in this article.

There are at least two different types of circuit asymmetry and lateralization to be discussed in the nervous system. The first type is on the circuits related to lateralized behaviors in the sensory and motor systems. Asymmetrically structured and/or functionally lateralized circuits in the sensory systems are involved in asymmetrically capturing information from the outer world, as is shown in the lateralized usage of the visual system in chicks. And asymmetrical or lateralized circuits of the motor systems may be involved in outputting information to the outer world in an asymmetric manner as lateralized behaviors. In the next section, which concerns the scale-eating cichlid fish in Lake Tanganyika, we review a current hypothesis regarding the evolution of lateralized behaviors. This hypothesis states that spatial asymmetry in environment and/or social interaction is thought to apply its natural selection pressure on the interfaces of sensory and/or motor circuits (Ghirlanda and Vallortigara, 2004; Ghirlanda et al., 2009). These include the lateralized predation behaviors of the scale-eating cichlid fish. Such lateralized behaviors are under the influences of predators, prey, or other individuals in the environment.

The second type is on the circuits without remarkable relation to lateralized behaviors. In the last three sections, we consider the evolution of the Hb, which is in the epithalamus of the diencephalon. The Hb has long been known to be asymmetrical in many vertebrates and is not related to lateralized behavior. Thus, one is faced with the difficulty of explaining how the Hb has evolved. We have two aims in this article. One is to point out that the current evolutionary hypothesis regarding behavioral lateralization is not applicable to the Hb. The other is to propose the new hypothesis that binary opposition in functional incompatibility applies selection pressure to the asymmetrical circuit of the Hb.

## Lateralized behaviors under the influence of social interactions in an evolutionary stable strategy: the example of the scale-eating cichlid fish in Lake Tanganyika

**Figure 2:**
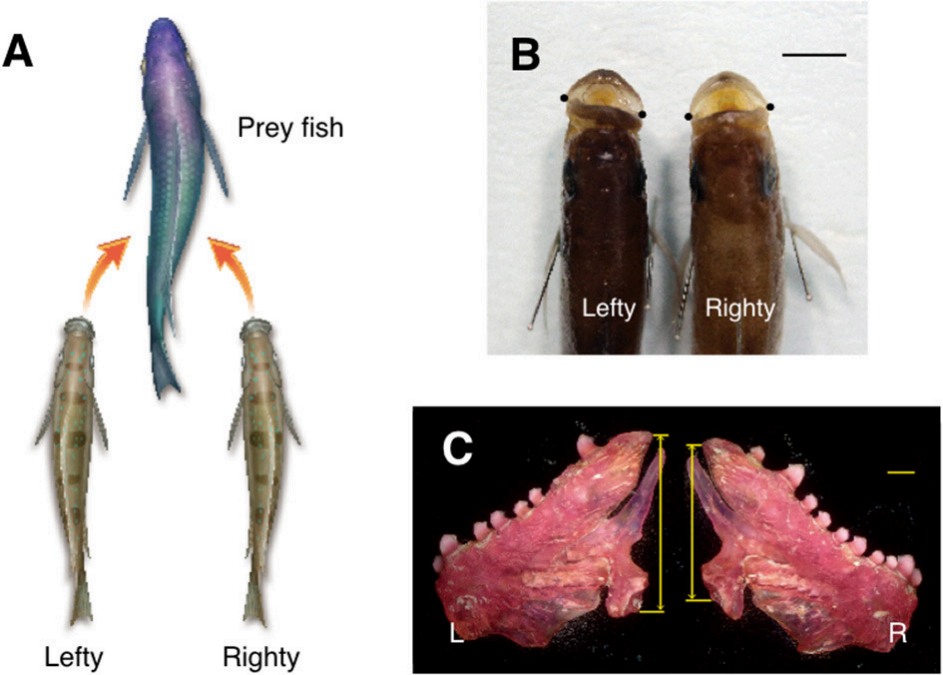
**Structural asymmetry and behavioral lateralization of the scale-eating fish, ***Perissodus microlepis*****. The behavioral lateralization in predation is shown. The lefty fish, with tilting mouth with larger left mandibular bone preferentially attack the left flank of the prey fish. The righty fish, with larger right mandibular bone have the opposite behavior **(A)**. The mouths of the scale-eating fish tilt to one side or the other with respect to the body axis. The dots indicate the lateral tip of the mouth in the dorsal view of head **(B)**. In the lefty scale-eating fish, the left mandibular bone is larger than the right. Arrows represent the height of the entire posterior dimension of the mandibular bone **(C)**. L, left; R, right. Scale bars, 1 cm **(B)**, and 1 mm **(C)**.

A scale-eating cichlid fish in Lake Tanganyika, *Perissodus microlepis*, exhibits lateralized behavior of predation (Hori, 1993). The cichlid fish is specialized to forage predominantly on the scales of the other fish (Fryer and Iles, 1972). About 50% of scale-eating cichlid fish in the field always attack and snatch scales from the left side of a prey (lefties), while the other fish attack the right (righties) (Figure 2A). The numbers of individuals in the lefty and the righty groups are not precisely equal, but are subtly different from each other at any point. The proportions of the lefty and righty fish oscillate around 50%, with a period of 4–5 years, and an amplitude of 15%. Thus, the proportions fluctuate between 65:35% and 35:65%. Because the prey eventually learns to predict the attacks of the cichlid fish on one side, cichlid fish in the majority have less of a chance to attack. This reduces the chance for the majority of the fish to survive and have offspring. On the other hand, individuals in the minority have a greater chance of attacking prey, as the prey does not expect an attack from the opposite side. This increases the chance for the minority to survive and have offspring, eventually. Thus, individuals of the minor phenotype are going to be more successful as predators than those of the major phenotype. Therefore, it has been suggested that the oscillations in the proportions of the lefty and righty fish are maintained by negative frequency-dependent selection (Hori, 1993; Takahashi and Hori, 1994). Although the lefty and righty fish are always competing with each other, this competition generates heterogeneity of lateralized behaviors in the species, which is stable in social interactions. This heterogeneity thus increases the chances for survival and contributes to the maintenance of the species as a whole. The lefties and the righties antagonistically interact with each other. In other words, prosperity of the majority (e.g., the lefty) is inhibited by the prosperity of the minority (e.g., the righty). This antagonistic interaction within the species influences the directional bias of lateralization to remain around 50:50. The results obtained from the studies in the scale-eating cichlid fish strongly support the theory of directional lateralization as an evolutionally stable strategy. This theory states that population bias of the direction is determined by the degrees of synergistic or antagonistic effects in the lateralized behaviors in social interactions (Takahashi and Hori, 1994).

Not only behavioral lateralization but also structural asymmetry is found in the mouth (Liem and Stewart, 1976) and the mandibular bone (Takeuchi et al., 2016), which is crucial for the success of the scale-eating (Figures 2B,C). In a recent report, structural asymmetry of the mouth is proposed to be inherited through multiple loci (Raffini et al., 2016). Thus, the asymmetrical body structure is under genetic control and its responsible genes are thought to be under natural selection pressure.

It is also expected that neuronal circuits responsible for the lateralized predation behavior of scale-eating are naturally selected by bilateral pressure from the outer world (disruptive selection; Rueffler et al., 2006) and exhibit asymmetrical structures and/or lateralized functions. However, a corresponding asymmetry has not yet been identified in neuronal circuits. The scale-eating fish approach their prey from a specific side. Thus, it is plausible that they use their visual system laterally, corresponding to the side they approach their prey. Subsequently, the lateralized predation behavior results from the output command of the motor system. Since the predation behavior is similar to the C-shaped escape behavior, their underlying circuits may be similar (Takeuchi et al., 2012). This possibility raises the chance to find an asymmetrical structure and/or a lateralized function in motor systems corresponding to the predation behavior. Therefore, the scale-eating fish could be an ideal field of study, pursuing neuronal mechanisms underlining lateralized behaviors.

Findings of lateralized behaviors in the cichlid fish strongly support the currently accepted hypothesis that the direction of behavioral lateralization is under the control of an evolutionally stable strategy (Ghirlanda and Vallortigara, 2004; Ghirlanda et al., 2009; Rogers et al., 2013). In this hypothesis, first, it is supposed that genes govern the generation of asymmetrical structures. Second, it is hypothesized that asymmetry or lateralization in the brain manifests as lateralized behaviors with left-right bias. Finally, it is thought that social interactions resulting from lateralized behaviors, such as interactions between individuals or between predators and prey, lead to natural selection pressures and generate population biases in asymmetry in and/or lateralization of the central nervous system. However, there are examples of structural asymmetry under the influences of natural selection pressure of social interactions in the absence of lateralized behaviors that cannot be explained by the current hypothesis. In the following sections, we consider these cases and propose a new hypothesis for the generation of asymmetrical circuits.

## Asymmetry of the habenula in zebrafish and hypothesis of binary opposite behaviors in functional incompatibility

**Figure 3:**
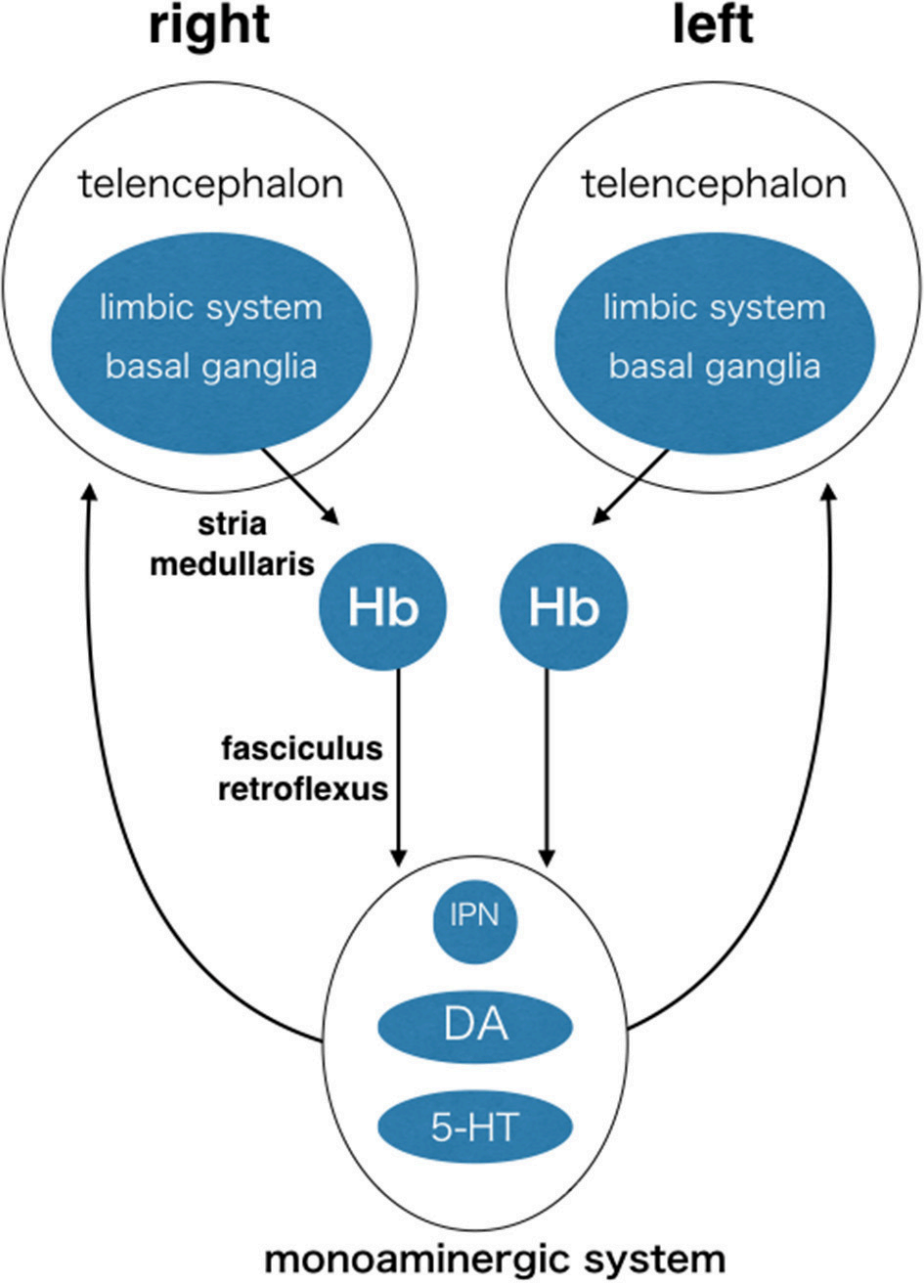
**The habenula (Hb) as a hub of circuit, where information is converged unilaterally and then diverged bilaterally**. Each side of the Hb receives inputs mainly from the limbic system and the basal ganglia through the same side of stria medullaris. The Hb sends outputs unilaterally and ventrally to the interpeduncular nucleus and the brain structures containing dopaminergic neurons and serotonergic neurons in ventral mesencephalon through fasciculus retroflexus. Monoaminergic nuclei send output diffusely and bilaterally to the many targets including the telencephalon. Many other connections are not shown, including reverse connections (e.g., from monoaminergic system to Hb) and commissural connections between the telencephalons (e.g., corpus callosum, anterior commissure, and posterior commissure). Hb, habenula; IPN, interpeduncular nucleus; DA, dopaminergic nuclei (e.g., ventral tegmental area and substantia nigra pars compacta); 5-HT, serotonergic nuclei (e.g., dorsal raphe and median raphe nuclei).

**Figure 4:**
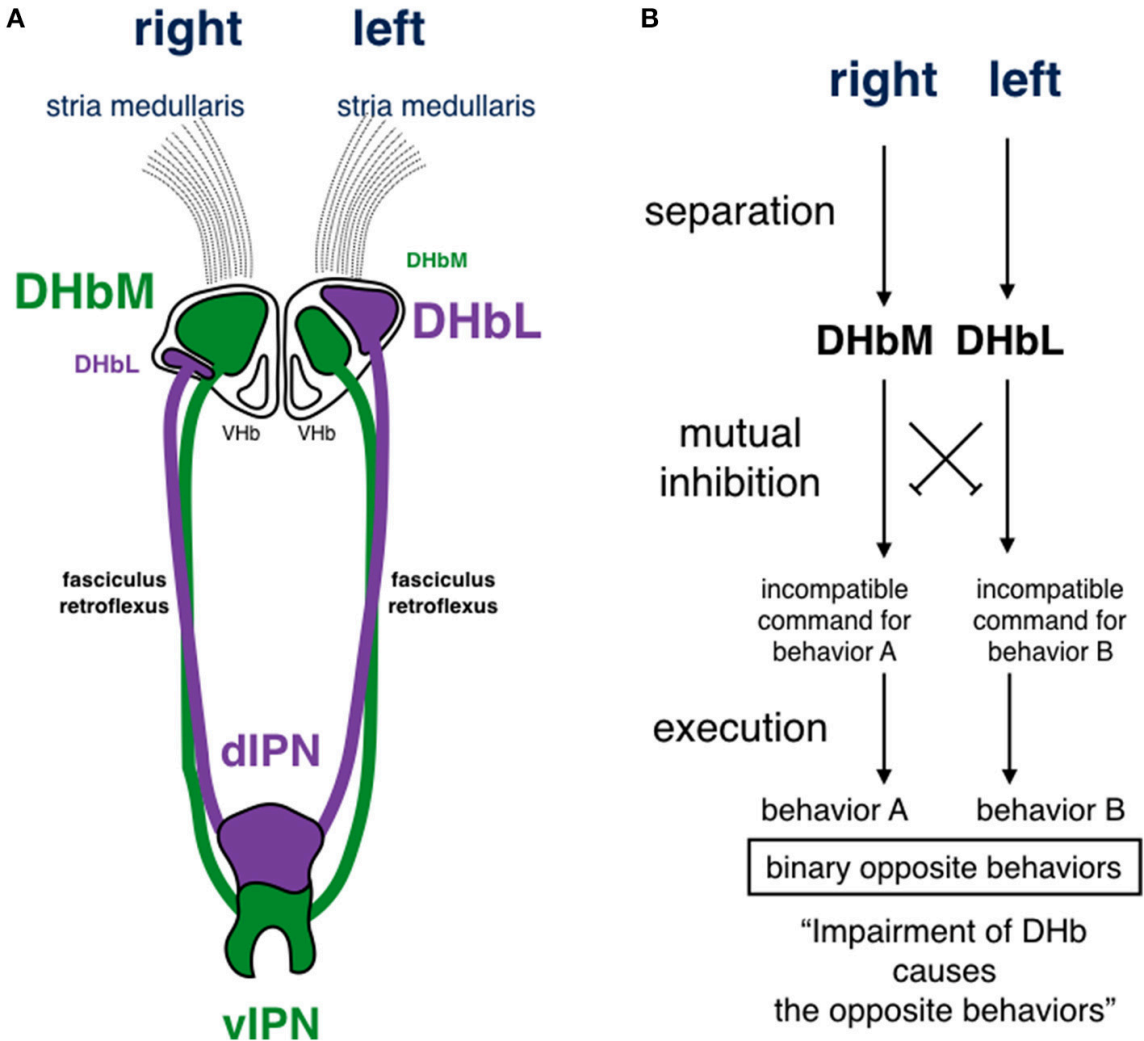
**Schematic drawing of habenular asymmetry and a circuit model for functional incompatibility in zebrafish. (A)** The dorsal habenula nucleus (DHb) is subdivided into medial and lateral subnuclei (DHbM and DHbL, respectively). The DHbM is larger on the right side than on the left side. The DHbL is larger on the left side than on the right side. They are connected to different parts of the interpeduncular nucleus (IPN). The DHbM is connected to the vIPN and the DHbL is connected to the dIPN. **(B)** There are two requirements for functional incompatibility: separation and mutual inhibition. In this model, the hypothetical mutual inhibition is supposed to be situated downstream of the DHb, where the impairment of the DHbM or the DHbL causes opposing behaviors. Because the DHbM and DHbL are asymmetrically separated on the right and left, the two circuits are asymmetrically structured on the right and the left. The experimental evidence by Agetsuma et al. (2010) and Chou et al. (2016) favor this model. DHb, dorsal habenular nucleus; DHbM, medial subncleus of dorsal habenular nucleus; DHbL, lateral subncleus of dorsal habenular nucleus; VHb, ventral habenular nucleus; dIPN, dorsal region of interpeduncular nucleus; vIPN, ventral region of interpeduncular nucleus.

The Hb is highly conserved in vertebrates. This suggests that its function is essential for survival and under influence of natural selection pressure common among different species from fish to mammals. The Hb is situated in the middle of the neuronal circuit between the telencephalon and the mesencephalon. It receives information concerning various aspects of emotion, such as failure, punishment, and stress, from the basal ganglia and the limbic system in the telencephalon through the stria medullaris unilaterally (Herkenham and Nauta, 1977; Matsumoto and Hikosaka, 2007). Axons derived from the Hb run unilaterally and ventrally, forming a pair of thick axonal bundles on both sides. Each of these bundles is called the fasciculus retroflexus (FR) and projects to midline nuclei in the mesencephalon (Herkenham and Nauta, 1979; Figure 3). Thus, the Hb is a hub wherein information conveyed to the unilateral Hb is relayed to nuclei that diverge bilaterally. This is likely to induce overall behavioral changes via bilateral projection systems, but is unlikely to cause lateralized behavior. In addition to its conserved structure, the Hb is well-known to exhibit structural asymmetry in many vertebrates (Concha and Wilson, 2001). In zebrafish, the Hb consists of two different nuclei, the dorsal and the ventral Hb (DHb and VHb; Concha et al., 2000, 2003; Gamse et al., 2003; Aizawa et al., 2005; Amo et al., 2010; Figure 4A). The DHb is composed of medial and lateral subnuclei (DHbM and DHbL). These subnuclei are connected topographically to different parts of the interpeduncular nucleus (IPN) in the ventral mesencephalon. The DHbM is connected to the ventral IPN (vIPN) and the DHbL is connected to the dorsal IPN (dIPN). The DHbM and the DHbL exhibit asymmetry between their left and right side. On the right side, the DHbM is large but the DHbL is small while on the left side, the DHbL is large but the DHbM is small. This asymmetry is with near 100% population bias except for the cases of situs inversus.

Various roles have been reported for the Hb (Brady and Nauta, 1955; Rausch and Long, 1974; Thornton and Bradbury, 1989). For example, the DHb is thought to be involved in the regulation of behaviors induced by fear. Expressing the tetanus toxin light chain specifically in the DHbL, Agetsuma et al. (2010) genetically inactivated the DHbL to study its role in behavior. In the fear-conditioning response, fish with inactivated DHbLs do not escape, but freeze instead, indicating that cessation of the escaping behavior is functionally linked to the generation of the freezing behavior. Thus, the DHbL is involved in the binary choice between escaping and freezing. However, it is not clear how the fish choose the alternative behavior of freezing instead of escaping when the DHbL is inhibited. Chou et al. (2016) inactivated the DHbL or the DHbM genetically and studied their effects on fighting behavior between two fish during a social conflict. Fish with inactivated DHbL tended to lose, even though their physical strengths, anxiety levels, locomotion activities, and aggressiveness were similar to those of wild type fish. On the other hand, fish with inactivated DHbM tended to win. These results indicate that information in the pathway involving the DHbL and the DHbM is involved in typical behaviors during social conflict. This suggests that the DHb functions in binary behaviors during social conflicts. The two experiments: fear-conditioning responses (escaping or freezing) and responses to aggression during social conflict (winning or losing), seem very different from each other. However, these experiments provide us with clues for understanding the role of DHb. The distinct behaviors are represented in the same nucleus. This indicates that the DHb is not involved in specific behaviors, such as escaping/freezing or winning/losing. Instead, it is plausible that the DHb is involved in mechanisms that are common between escaping/freezing and winning/losing, where both sets of behaviors are in binary opposition. The fish cannot perform these behaviors simultaneously, but must choose one behavior over the other. Impairment of the DHbL during fear-conditioning caused the fish to choose freezing instead of escaping. During social conflict, impairments of the DHbL and the DHbM made the fish to perform opposing behaviors; DHbL impairment led to losing, and DHbM impairment led to winning. Agetsuma et al. (2010) and Chou et al. (2016) likened this aspect of the DHb to that of a switchboard. The DHb is not involved in lateralized behaviors. Its role is neither similar to the sensory circuits for the asymmetrical usage of the eye in birds (Rogers and Anson, 1979; Rogers, 1982, 2000; Sandi et al., 1993), nor to the motor circuits assumed in the lateralized behaviors of scale-eating cichlid fish (Hori, 1993; Takahashi and Hori, 1994; Takeuchi et al., 2012, 2016). Unlike sensory and motor circuits, the DHb is likely to participate in the circuit processing binary opposite behaviors that are functionally incompatible.

The term “functional incompatibility” means that the mechanisms adequate for the solution of one problem are incompatible with those needed to resolve another problem (Rogers et al., 2013). The incompatibility between escaping and freezing prevents the fish from swimming against threats, which is beneficial for their survival. The incompatibility between winning and losing behaviors causes the fish to avoid fatal fights and enables them to establish a social hierarchy, which is beneficial for the survival of each individuals and maintenance of the group. Thus, it is reasonable that these incompatibilities are favored by natural selection. The structures and functions of neuronal circuits for binary opposite behaviors are thought to be under natural selection pressure resulting from functional incompatibility. The functional incompatibility among logical demands is hypothesized to underlie the evolution of multiple systems in biology (Sherry and Schachter, 1987).

There would be two requirements for making the animals to engage in incompatible behaviors. The first requirement would be separation; the incompatible behaviors are processed separately in each circuit. The second would be mutual inhibition; one circuit inhibits the other and vice versa. Thus, it is thought that a set of separated circuits mutually inhibiting each other has evolved in organisms (Figure 4B). In the first requirement, functional incompatibility may be separated through asymmetry during evolution. From a computational point of view, it has been suggested that segregation of functions in the halves of the brain is likely to enhance ability of information processing by decreasing its circuit path and reduce the cost of duplicating circuits, resulting in greater benefits for survival. This represents a solution to the problem of functional incompatibility (Vallortigara et al., 1999), and such concept was confirmed by experimental evidences in zebrafish (Agetsuma et al., 2010; Chou et al., 2016). Indeed, the incompatible behaviors of escaping/freezing and winning/losing are processed in the asymmetrical left and right nuclei of the DHb. Thus, it is very likely that the DHb is the nucleus representing functional incompatibility and that its asymmetrical structure ensures separation of the binary opposition. One may then ask where the DHb is placed in the circuits of functional incompatibility and what its role is.

In the upstream circuits of the DHb, information from the environment, such as threats or social conflict, is assessed in the sensory systems, the amygdala, and the extended amygdala. The inactivation of the extended amygdala in mice (the bed nucleus of the stria terminalis) resulted in an ignorance of the smell of a predator, and the mice did not escape or freeze. This indicates that the bed nucleus of the stria terminalis is involved in the early steps in the processing of functional incompatibility (Kobayakawa et al., 2007). It is thought that the information is processed differentially based on its importance (e.g., predator or not) and amount (e.g., intensity of the smell). This is then thought to result in the binary outputs inducing incompatible behaviors. Because the DHb receives fibers of the stria medullaris unilaterally, the information is thought to be separately lateralized in upstream structures before arriving at the DHb. The functional lateralization might be generated between the extended amygdala and the Hb. Thus, there is a possibility that the binary opposite behaviors are generated not only in the DHb but also in the circuit chain to the DHb. To examine this possibility, it would be informative to look for functional lateralization in the upstream of the Hb. In addition, because the DHb is involved in incompatible behaviors and is situated in the upstream of mesencephalic nuclei, such as the IPN and the monoaminergic nuclei, which modulate various aspects of behavior, it is thought to participate in circuits near to the execution of binary opposite behaviors.

As for the second requirement, mutual inhibition must occur downstream of the DHb because genetic inactivation of the DHb resulted in alternative behaviors in binary opposition (escaping/freezing and winning/losing) (Figure 4B). However, the mutual inhibition has not yet been found in pathways involving the DHb. Although Chou et al. (2016) indicated that there is no neural connections between the DHbL and the DHbM or between the dIPN and the vIPN, the connection should be hidden in other unknown place.

Taking all of the above into consideration, we propose the hypothesis of binary opposition in functional incompatibility to explain the evolution of asymmetry in the Hb. Even without lateralized behaviors, the asymmetrical Hb is thought to have evolved under the natural selection pressure resulting from functional incompatibility. Because it is essential for survival and under the common selection pressure, the asymmetrical Hb is thought to be broadly conserved in vertebrates. In zebrafish, Nodal signaling plays pivotal roles in determining the direction of the Hb asymmetry (Concha et al., 2000, 2003; Aizawa et al., 2005; Carl et al., 2007; Inbal et al., 2007).

While Hb asymmetry is conserved, directions, and degrees of its asymmetry differ among species (Concha and Wilson, 2001). Villalón et al. (2012) analyzed interspecies differences in DHb asymmetry in seven species of teleost fish, although they did not differentiate between the DHbM and the DHbL. In all species examined, the DHb exhibited asymmetry. This again indicates that the selection pressure of functional incompatibility has been commonly and consistently exerted on the DHb and made this structure to be asymmetrical. However, the direction of its asymmetry varies among different species. In *Danio rerio* (*D. rerio*, zebrafish), *Epalzeorhynchos bicolor* (*E. bicolor*, redtail sharkminnow), *Oryzias latipes* (*O. latipes*, medaka), *Poecilia reticulata* (*P. reticulata*, guppy), and *Betta splendens* (*B. splendens*, Siamese fighting fish), the left DHb is larger than the right. The left-right difference is more remarkable in *D. rerio* and *E. bicolor*, but is moderate in *O. latipes*. This shows that left side bias has varying strengths in population asymmetry. In *Fundulopanchax gardneri* (*F. gardneri*, Steel-blue Killifish) females, the right DHb is larger than the left, which indicates a right side bias. Additionally, in *F. gardneri* males and in *Pterophyllum scalare* (*P. scalare*, angelfish), one side of the DHb is larger than the other, although the larger side is different in each fish. This indicates individual asymmetry with no population bias.

The mechanism by which the direction of DHb asymmetry is determined remains unknown. However, we propose the following two hypotheses. One is based on a stochastic idea, which states that the direction is under the influence of genetic drift (the hypothesis of genetic drift about the direction of Hb asymmetry). Genetic drift leads to fluctuations in gene frequencies affecting Hb asymmetry, making either side of the Hb dominant with no advantage nor disadvantage for survival (Lande, 1985; Barton and Rouhani, 1987). This fluctuating asymmetry is thought to be stabilized in the group because of the low probability of its reversal. This may lead to interspecies differences in the direction of the asymmetry. The other hypothesis is based on a concept of natural selection that selection pressures resulting from functional incompatibility determines the directions. Even though the directions are diverse, laterotopic projections are conserved; the left DHb projects to the dorsal IPN, and the right DHb projects to the ventral IPN (Villalón et al., 2012). This indicates that information passageways are kept separated and are wired to the same downstream targets. The conserved wiring enables the organisms to process information for binary opposite behaviors in a similar manner. Assuming that the subnuclear organization of the DHb is common between *D. rerio* (zebrafish) and other species, the interspecies differences in the directions suggest that the dominances of the DHbL or the DHbM vary among the species. This may indicate that behaviors in binary opposition (e.g., escaping/freezing and winning/losing) do not occur over the same threshold, but are displayed under different thresholds in each species, leading to differential sociability (Bisazza et al., 2000). In this sense, the questions proposed by Villalón et al. (2012) and Chou et al. (2016) are highly valuable, whether the directions of the DHb asymmetry are related to differences in sociability among the species or not. Therefore, not only the asymmetry itself, but also the directions, the degrees, and the strengths of the asymmetry may be evolved under the selection pressure resulting from functional incompatibility (the hypothesis of binary opposite behaviors threshold about the direction of Hb asymmetry). This may also indicate that diversity of the directions among the species is under the influences of selection pressures different in social interactions and/or environments. Both the genetic drift hypothesis and the natural selection hypothesis can be examined experimentally in the future (Lamichhaney et al., 2016).

## Symmetry of the habenula in mice and its lateralization

In spite that basic cytoarchitecture of the Hb is conserved, the mammalian Hb shows structural symmetry exceptionally (Concha and Wilson, 2001). The mammalian Hb consists of the medial (MHb) and lateral habenular nuclei (LHb), which are homologous to the zebrafish DHb and VHb, respectively (Amo et al., 2010). Similar to zebrafish, the Hb axons form the FR. In the FR, the axons from different origins are topographically organized. The axons from the MHb run in the core of the FR and project to the IPN. On the other hand, the axons from the LHb run in the sheath of the FR (Herkenham and Nauta, 1979; Ichijo and Toyama, 2015), sending outputs to outside the IPN: the dopaminergic (DA) and the serotonergic (5-HT) nuclei.

Using a simple transgene with a long half-life fluorescent protein (Venus) under the control of an immediate early gene (*zif268/egr1*) promoter, we labeled the history of neuronal activity and found that the LHb is functionally lateralized in mice (Ichijo et al., 2015). During the immature stage around postnatal day 13 (P13), the sheath of the FR was unilaterally labeled by the history of neuronal activity in the LHb. The unilateral labelings in the FR were observed up to P20, but not after P35. Expression of intrinsic ZIF268/EGR1 proteins in the LHb was lateralized around P13. Thus, activation of the LHb induced by stress caused unilateral labeling of the FR. The lateralization was not biased to either side at the population level (left, 45.8%; right, 54.2%; *n* = 72). In addition, there was no sexual difference between males and females (male: left, 45.9%; right 54.1%; *n* = 37; female: left, 45.7%; right 54.3%; *n* = 35). Thus, the symmetrical LHb is functionally lateralized without directional bias or sexual differences during postnatal development and in the stress response in mice.

**Figure 5:**
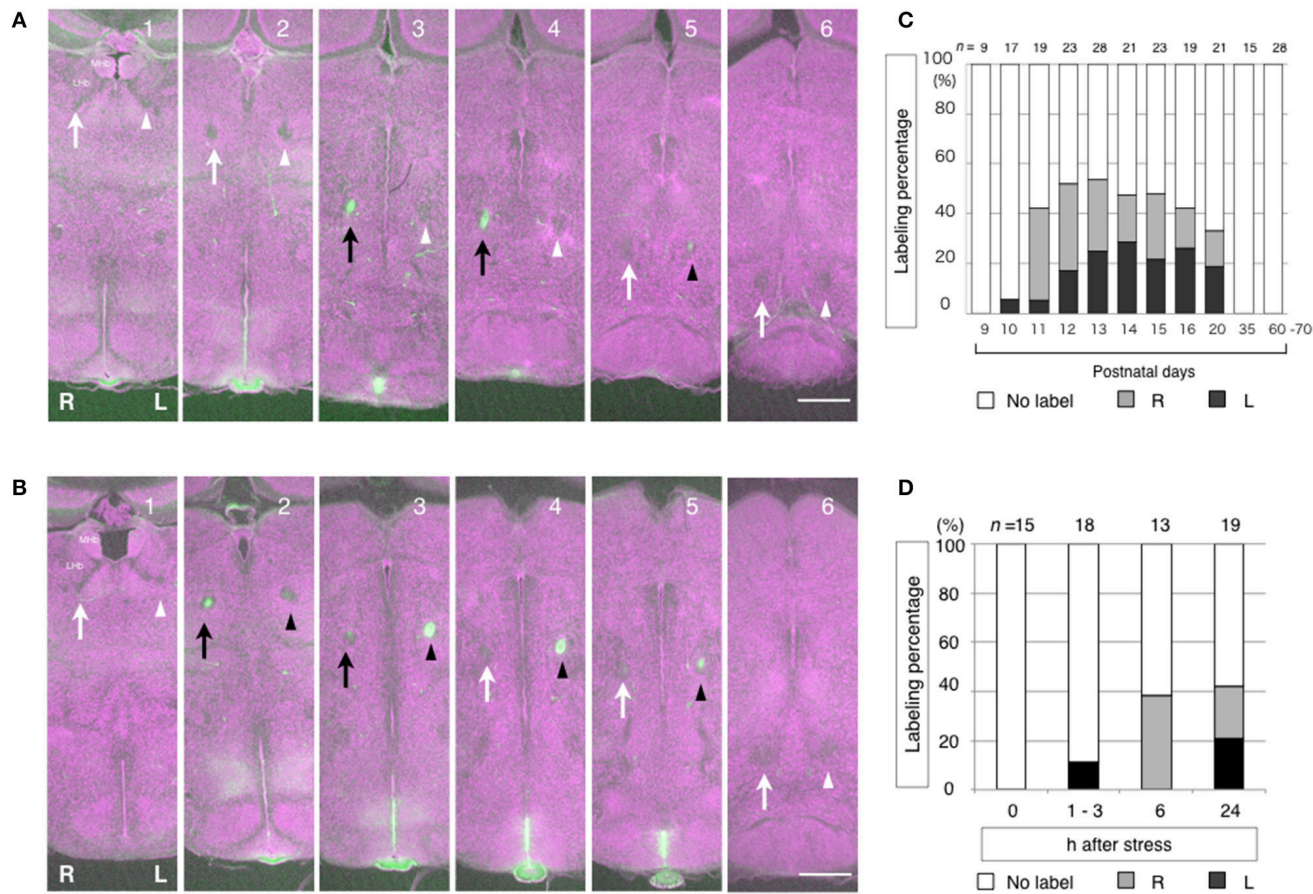
**Alternate labeling and temporal shift of the labeling in between the left and the right fasciculus retroflexus (FR) reflecting the history of LHb neuronal activity**. The segments of the FR are labeled alternately between the right and left at P11 **(A)** and P13 **(B)**. In case A (P11), the right FR is dominantly labeled. Dorsoventral courses of the FR are shown in successive coronal sections of mouse diencephalon from the anterior (1) to the posterior (6). The history of neuronal activity is labeled in green. Cytoarchitecture is visualized in purple by the fluorescent Nissl stain. The right FR is indicated by arrows. The left FR is indicated by arrowheads. The labeled FRs are indicated as black arrows and black arrowheads, on the right and left, respectively. In case B (P13), the left FR is dominantly labeled. During development, the right FR is dominantly labeled in the early phase at P11 and P12. Then, the left FR labeling appears in the late phase at P13 and thereafter **(C)**. In responses to stress, the right FR is dominantly labeled in the early phase, which is 6 h after stress. Then, the left FR labeling appears in the late phase, 24 h after stress **(D)**. MHb, medial habenular nucleus. LHb, lateral habenular nucleus. R, right; L, left. Scale bars, 500 μm. **(C,D)** Ichijo et al. (2015) with permission from Elsevier.

Careful examinations of the labelings showed that one side of the FR was not exclusively labeled. Instead, one side was more intensely labeled than the other side. Furthermore, neither side of the FR was equally labeled in any of the mice examined. The segments of the FR were often labeled alternately between the left and right FR along the dorsoventral axis (Figures 5A,B). Because of the growth associated protein-43 (GAP-43) membrane localization sequence linked to Venus, the labeling was localized to the cell surface. Generally, in axon tracing experiments using lipophilic membranous tracers, if given enough time, a large amount of the tracers labels entire neurons from the soma to axon terminals. However, a small amount only labels parts of the axons (Ichijo, 1999; Ichijo and Toyama, 2015). Therefore, in our experiments, the LHb neurons may have transiently produced an insufficient amount of Venus protein that is only enough to label segments of the FR. In addition, there is a tendency that the right FR is labeled in the early phase, while left FR labeling appears to occur in the late phase. The shift of the labeling between the right and the left from the early to the late stages is common during development and in response to the stress. The right FR is labeled at P11 during development and at 6 h after stress. Left FR labeling appears at P13 during development and at 24 h after stress (Figures 5C,D). The observations of: (1) spatial alternations in labeling of the FR segments between the right and left, and (2) the temporal shift of the labeling from early in the right to late in the left are suggestive of oscillation. In the mouse, the direction of lateralization of the LHb may not be restricted to one side or the other. Instead, the LHb may be dynamically activated between the right and the left during the time course of its activation. It is thus possible that LHb lateralization is caused by oscillatory activation between the right and the left. Further functional investigations would be informative in the study of the nature of LHb lateralization. Until recently, functional lateralization of the mammalian Hb had not been described. However, a fMRI study in humans has indicated that the Hb is lateralized. Specifically, the activities of the left and the right Hb were shown to be not correlated and the left and the right Hb were shown to be functionally connected to different brain areas, although the MHb and the LHb were not distinguished (Hétu et al., 2016).

The Hb receives information about cognition and emotion related to the external and internal states of the animal. Such information includes sensory/motor, reward, arousal, and emotion/stress data. This information originates mainly in the basal ganglia and the limbic system in the telencephalon and is projected to the Hb through the unilateral stria medullaris (Herkenham and Nauta, 1977; Araki et al., 1984; Figure 3). From this lateralized input, the LHb integrates the information and provides a passageway to monoaminergic neuromodulator systems in the ventral mesencephalon. It sends outputs to the DA nuclei of the ventral tegmental area and the substantia nigra pars compacta through the rostromedial tegmental nucleus. It also sends outputs to the 5-HT nuclei of the dorsal and median raphe nuclei (Herkenham and Nauta, 1979; Araki et al., 1988; Yañez and Anadón, 1996; Jhou et al., 2009; Kaufling et al., 2009; Figure 3). They diverge information bilaterally, influencing functions of broad targets, influencing the choices for relevant adaptive behaviors, and switching between goal-directed behaviors (Baker et al., 2015). The information conveyed to the unilateral LHb is likely to induce behavioral changes through the bilateral projection systems of the DA and 5-HT, but unlikely to cause lateralized behavior. Because the LHb receives input from the unilateral stria medullaris, upstream circuits must be already functionally lateralized.

When LHb activity is regulated, animals are typically confronted with opposing behavioral choices, such as moving to seek rewards or not moving to avoid negative consequences. During such decision making, the LHb regulates target nuclei in the DA and 5-HT systems (Matsumoto and Hikosaka, 2007; Hikosaka, 2010). The LHb seems to have general roles in behavioral choices. Baker et al. (2015) proposed that, when the switching of behavioral strategies is required, the LHb plays a role in the execution of goal-directed behaviors and is involved in behavioral flexibility. In their proposal, aimed at receiving rewards or avoiding punishments, the LHb signals information about the ongoing behavioral state to organize adaptive actions in monoaminergic systems. This proposal is worth considering because the DA and 5-HT systems interact with each other in a complimentary fashion during behavioral flexibility, such that balanced increases in DA and 5-HT levels are correlated with ideal reversal learning performance in the orbitofrontal cortex and the striatum (Doya, 2008; Robbins and Arnsten, 2009; Bari et al., 2010; Groman et al., 2013; Liu et al., 2014). Therefore, it is rational to consider that the LHb is involved in behavioral choices in a flexible manner, as the LHb is not asymmetrically structured, but is functionally lateralized.

## Differences in evolutionary strategies in the habenula between zebrafish and mice: asymmetry vs. lateralization

Zebrafish and mice are thought to have adopted different strategies for their survival in the Hb structures. The asymmetry of the zebrafish DHb is thought to be a structural representation of functional incompatibility, where the asymmetrical DHb causes highly programmed behavioral choices in binary opposition. This enhances ability of information processing by decreasing the circuit path. Moreover, this prevents circuits from being duplicated, reducing costs, and contributing to an increase in survival. Therefore, zebrafish are thought to have adopted a structurally fixed strategy. From the circuit point of view, because asymmetrical circuits in the DHb are structured in all zebrafish, all are likely to exhibit stereotyped behaviors in binary opposition. This leads to higher reproducibility in individual behaviors of fish organized in the group, producing consistent social interactions. From the social point of view, because incompatible behaviors are highly organized, the social interactions between zebrafish are thought to be relatively simple, consistent, and rigid with little room for adjustment. This may lead to selection pressure on the fixed circuits and result in an asymmetrically structured DHb.

In contrast, mice are thought to have adopted a functionally flexible strategy, which enables them to perform variable behaviors and possibly produce flexible social interactions. From the circuit point of view, the lateralized usage of the symmetrical LHb makes it possible to process complicated information for goal-directed behavioral choices. Various information in environment is likely to be used for the estimation of reward-prediction errors, which could cause variable behavioral choices in opposition. Because the LHb is functionally lateralized in every mice, each mouse is likely to exhibit variable behavior depending on the circumstances. This would lead to the control of individual behaviors in the group (Matsumoto and Hikosaka, 2007; Hikosaka, 2010). Thus, it is hypothesized that the lateralization of the LHb is important in dynamic regulation of behavioral choices. Functional regulation of behavioral choices is beneficial for mice because it increases their survival, even though it leads to the increased costs of circuit duplication. From the social point of view, because the behaviors are variable, the social interactions of the mice fluctuate, which may lead to selection pressure on circuits that are adjustable. As a result of complicated environment in which mice suffer, the LHb is functionally lateralized instead of having structural asymmetry.

For future researches, it is worth verifying whether structural asymmetry vs. functional lateralization of Hb circuits are related to stereotyped vs. flexible behaviors in individuals, and rigidity vs. flexibility in social interactions in the group.

## Ethics statement

Animal experiments were carried out in accordance with the National Institute of Health Guide for the care and use of laboratory animals. All experimental protocols were approved by the Committees for Animal Care and Use of the University of Toyama (A2013MED-19, A2015MED-47). All efforts were made to minimize the number of animals used and their suffering. The scale-eating cichlid *Perissodus microlepis* is widely distributed in Lake Tanganyika. The species is not protected all up until now (refered FishBase http://www.fishbase.org/summary/8801).

## Author contributions

HI wrote the manuscript and made the Figures 1–5. TN made Figure 1. MK made Figure 3. YT made Figure 2.

### Conflict of interest statement

The authors declare that the research was conducted in the absence of any commercial or financial relationships that could be construed as a potential conflict of interest.

## Abbreviations

Hb: habenula
FR: fasciculus retroflexus
DHb: dorsal habenular nucleus
VHb: ventral habenular nucleus
DHbM: medial subnucleus of DHb
DHbL: lateral subnucleus of DHb
IPN: interpeduncular nucleus
vIPN: ventral region of IPN
dIPN: dorsal region of IPN
MHb: medial habenular nucleus
LHb: lateral habenular nucleus
DA: dopamine
5-HT: serotonin.
